# World no-tobacco: effects of second-hand smoke (SHS) and vapors on the developing and adult brain

**DOI:** 10.3389/fphar.2025.1466332

**Published:** 2025-03-06

**Authors:** Glen Kisby, Jacob Raber

**Affiliations:** ^1^ Department of Biomedical Sciences, College of Osteopathic Medicine of the Pacific Northwest, Western University of Health Sciences, Lebanon, OR, United States; ^2^ Department of Behavioral Neuroscience, Oregon Health and Science University, Portland, OR, United States; ^3^ Departments of Behavioral Neuroscience, Neurology, Psychiatry, and Radiation Medicine, Division of Neuroscience ONPRC, Oregon Health and Science University, Portland, OR, United States

**Keywords:** environmental tobacco smoke (ETS), nicotine, oxidative stress, e-cigarettes, DNA damage/repair

## Abstract

The goal of this review is to highlight the role of second-hand smoke (SHS) or environmental tobacco smoke (ETS) and e-cigarette (EC) vapors on brain integrity and function during development and adulthood, including how it relates to increasing the risk for age-related neurodegenerative disorders. A systematic review of the literature of the effect of SHS or ETS and e-cigarette vapors on the brain revealed a total of 284 or 372 publications and 312 publications, respectively. After taking into account duplicate publications or publications focused on policy, surveys or other organs than brain, there are limited studies on the effects of SHS, ETS or EC vapors on brain structure and function. In this review, we examine the major constituents in SHS or EC vapors and their effects on brain health, mechanisms by which SHS or vapors alters brain integrity and function, including behavioral and cognitive performance. We hope that this review will encourage investigators to explore further the short-as well long-term effects of SHS or vapor exposure on the developing and adult brain to better understand its role in neurodevelopmental disorders and neurodegenerative diseases and ultimately to develop therapeutic modalities to reduce or even prevent the short- and long-term detrimental effects on brain health.

## 1 Introduction

**FIGURE 1:**
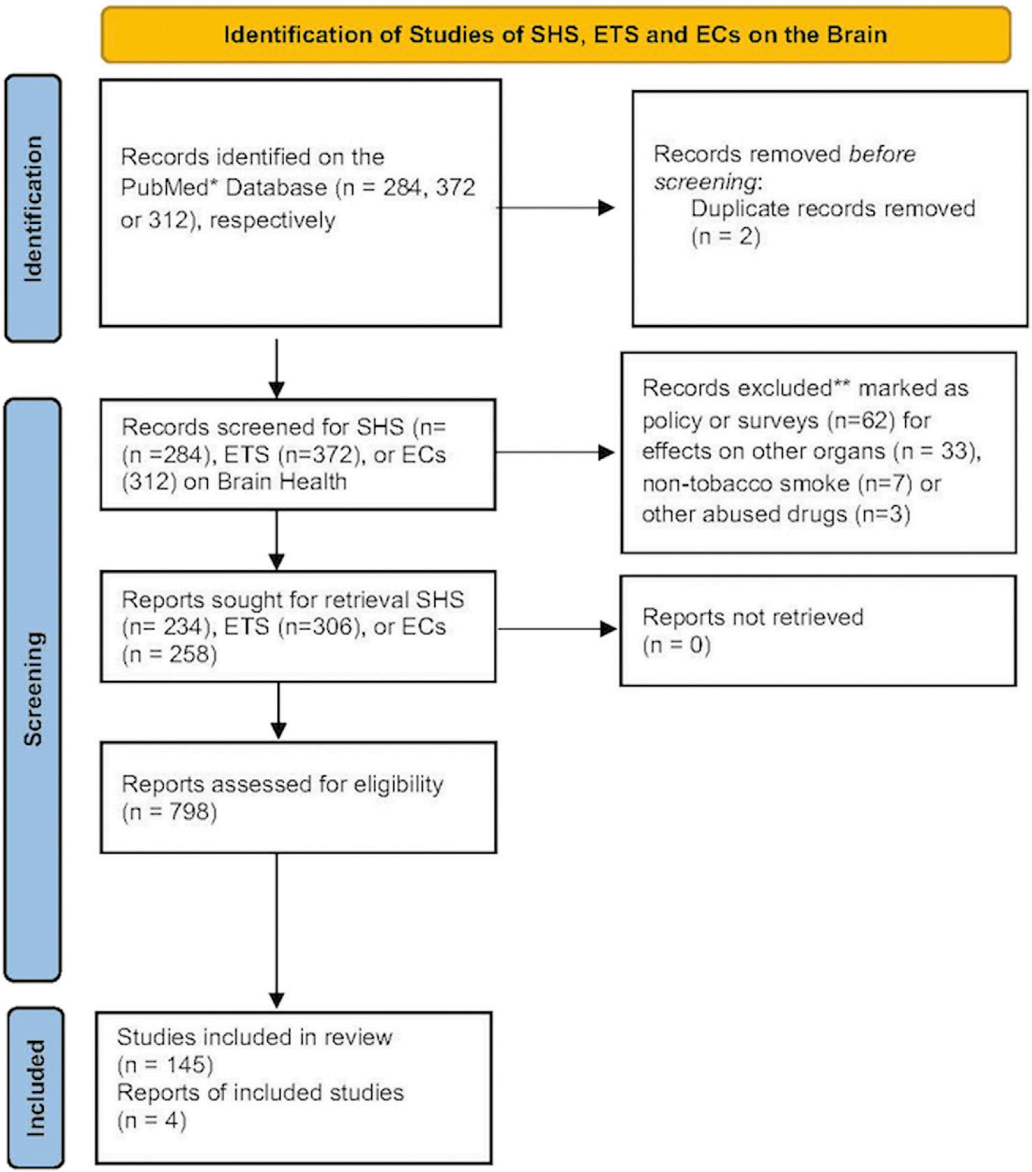
The number of records identified from the PubMed database was searched using the terms SHS, ETS or EC and brain. Records that were excluded from this review were those focusing primarily on policy or surveys, effects on non-nervous tissues, non-tobacco smoke or from other abused drugs. These reduced the number of records for each term that focused primarily on brain health. A document from the CDC on a visual dictionary of electronic cigarettes or vaping was also included in this review (Centers for Disease Control and Prevention, 2024).

Please see Figure 1 for the Prisma statement.

Tobacco use is one of the leading risk factors for disease burden and mortality worldwide, contributing to 229.8 million (95% uncertainty interval: 213.1–246.4 million) disability-adjusted life years and 8.7 million (8.1–9.3 million) deaths in 2019 (Flor et al., 2024). Second-hand smoke (SHS) exposure, also referred to as passive or environmental tobacco smoke (ETS), is a major tobacco-related public health concern for nonsmokers. SHS increases the risk of nine health outcomes, including ischemic heart disease stroke, diabetes and lung cancer, but the effects on the nervous system have not been extensively examined (Flor et al., 2024). Although smoking rates have gradually declined over the past 50 years, ∼37% of the global population is still being exposed to the smoke emitted from the burning end of tobacco products (sidestream smoke, SS) or exhaled from smokers (mainstream smoke, MS), with higher rates of exposure reported among women and children compared to men (Flor et al., 2024). This is concerning, since tobacco smoke is composed of thousands of toxic chemicals and compounds, including many carcinogens, which when inhaled can lead to disease and death, especially among vulnerable populations.

SHS or ETS consists of sidestream (∼85%) and mainstream smoke (∼15%) (Soleimani et al., 2022). Mainstream smoke is exhaled from a smoker, while sidestream smoke is smoke emitted from a burning cigarette. Indoors, SHS can persist for hours to become more toxic with time and duration, a process known as aging (Schick and Glantz, 2005; Schick and Glantz, 2007). When second-hand smoke is released into the open air, it changes both chemically and physically. Human exposure to SHS depends on airflow patterns, dilution volume, the distance between smokers and non-smokers, and smoking prevalence (Public Health Service, 2006). The toxic chemicals in SHS can also react with atmospheric air to generate other toxins that can be inhaled (Centers for Disease Control and Prevention, 2010; Fu et al., 2012). SHS contains >7,000 chemicals with at least 70 of them being carcinogenic (Li and Hecht, 2022). Nicotine, polycyclic aromatic hydrocarbons (PAHs) and aldehydes (formaldehyde, acetaldehyde and propionaldehyde) are the most abundant chemical constituents commonly found in SHS. SHS also contains smaller amounts of metals and nitrosamines (Soleimani et al., 2022). However, aldehyde concentrations in SHS can exceed those of nicotine, depending upon exposure conditions (*e.g.*, indoor vs outdoor places). PAHs, formaldehyde, and acetaldehyde all have the potential to damage DNA (genotoxins) associated with neurodevelopmental and neurological disorders (Perera et al., 2014; Joshi et al., 2019; Rana et al., 2021; Kou et al., 2022; Yuan et al., 2023). These genotoxic effects might also vary among different ethnic populations. For example, recent studies indicate that a mutation of the enzyme that metabolizes formaldehyde and acetaldehyde (aldehyde dehydrogenase 2, ALDH2) is highly prevalent (30%–50%) among the East Asian population (Chen et al., 2015; Wang X. et al., 2024), which can increase the susceptibility to second-hand exposure to toxins (Ma et al., 2024).

Electronic cigarettes (e.g., e-cigarettes, ECs, vape pens, etc.) or electronic nicotine delivery systems (ENDS) contain a liquid solution (“pods”) composed of various amounts of nicotine, flavoring substances, and other chemicals that is vaporized upon activation of an electronic heating element that is triggered by inhalation (Grana et al., 2014; Lopez-Ojeda and Hurley, 2024). Using ECs, is commonly known as ‘vaping. Details about the different types of ECs, their components and aerosols can be found in recent reviews by the Center for Disease Control (Centers for Disease Control and Prevention, 2024) and other investigators (Lopez-Ojeda and Hurley, 2024; Omaiye et al., 2022; Heywood et al., 2024). ECs have been promoted as containing fewer toxic chemicals than conventional cigarettes suggesting that these type of cigarettes are less harmful (Heywood et al., 2024; Hamann et al., 2023; Izquierdo-Condoy et al., 2024). However, the exhaled vapors from e-cigarettes also contains toxic chemicals and carcinogens (*e.g*., acrolein, benzene, diacetyl, formaldehyde) following second-hand exposure of individuals (Lopez-Ojeda and Hurley, 2024; Armendariz-Castillo et al., 2019; Yan et al., 2021). These findings suggest that e-cigarettes may be less harmful for smokers, but they are not safe for non-smokers (Izquierdo-Condoy et al., 2024). While there are many studies examining the neurological effects of e-cig vapors in humans (Hamann et al., 2023), there have been limited studies to investigate the direct effects of e-cigarette vapors on brain function and structure using animal models (Siegel et al., 2022; Ruszkiewicz et al., 2020). The vapors from e-cigarettes also contain significant amounts of nicotine, as well as other constituents found in SHS, but at lower concentrations (Ebersole et al., 2020) (Table 1). Formaldehyde can also be formed during the partial combustion of propylene glycol and glycerol liquids in e-cigarettes (Strongin et al., 2024), which reaches more deeply into the lungs than gaseous formaldehyde (Pankow, 2017).

**TABLE 1:** Chemical components found in SHS from Conventional and Electronic cigarettes.

Chemical	^a^Conventional	^b^Electronic (E-cigs)
Nicotine^c^	0.85–100 μg/m^3^ (6.80 μg/m^3^)	0–50 mg/mL
Formaldehyde	49 μg/m^3^	0–11 µg/10 puffs
Acetaldehyde	1,390 μg/m^3^	0–4.5 µg/10 puffs (341 μg/m^3^)
Propionaldehyde	120 μg/m^3^	ND (87 μg/m^3^)
Acrolein	2.3–275 μg/m^3^	0–1.0 µg/10 puffs
AA	43.43–155.11 ng/m^3^	147 ng/m^3^
Metals (Cd, Cr, Ni)	0.03–0.01 μg/m^3^ 0.0012–0.009 μg/m^3^ 0.0025–0.007 μg/m^3^	ND 0.0846 mg/m^3^ 0.04 mg/m^3^
Nitrosoamines (NNN, NNK, NDMA, NPYR)	ND – 0.006 μg/m^3^ ND – 0.0135 μg/m^3^ 0.008–0.045 μg/m^3^ 0.0025–0.007 μg/m^3^	ND – 0.06 ng/g ND – 0.06 ng/g Not determined Not determined

^a^ Adapted from Arfaeinia et al. (2023).

^b^ Nicotine varied depending on the source of exposure (i.e., indoor air, restaurants, bars or discotheques). Mean concentrations in parentheses were from Soleimani et al. (2022).

^c^ Adapted from multiple sources (Goniewicz et al., 2018; Vivarelli et al., 2024; Farsalinos and Gillman, 2017; Schober et al., 2014; Olmedo et al., 2018; Quintana et al., 2021). ND, below detection limits.

Exposure to second-hand smoke (SHS) at different times during brain development (fetal, infant, and adolescence) can produce short- or long-term effects on brain structure and function leading to neurodevelopmental disorders (NDDs) (Slotkin et al., 2015; Lin et al., 2021; Ou et al., 2024). Children exposed from pregnancy to childhood have a higher risk of developing Attention Deficit Hyperactivity Disorder (ADHD) during school-aged years and this risk is somewhat stronger for SHS exposure during the prenatal and postnatal periods (Lin et al., 2021). A more recent study of SHS exposure and neurodevelopmental disorders also revealed that exposure is associated with higher risk of ADHD and other learning disabilities (Ou et al., 2024). Collectively, these studies demonstrate that SHS can induce short- and long-term structural effects on the developing brain to cause permanent functional changes. The contribution of individual toxins in cigarette on both brain development and function appears to be greater when they are combined (Slotkin et al., 2019). Combined exposure of pregnant rats to both a PAH (*i.e.*, benzo [a]pyrene) and nicotine impairs acetylcholine presynaptic activity and upregulates acetylcholine and serotonin receptors in adolescent rats when compared with exposure to either agent alone. These studies demonstrate that exposure to combinations of SHS chemicals is more detrimental to the developing brain than single exposure to SHS chemicals. Since SHS contains more than 4,000 chemicals (Arfaeinia et al., 2023), two or more of them may be more detrimental to the developing brain, but studies assessing combinations of SHS chemicals have yet to been conducted.

## 2 Pharmacology and pathology of nicotine in the brain

**FIGURE 2:**
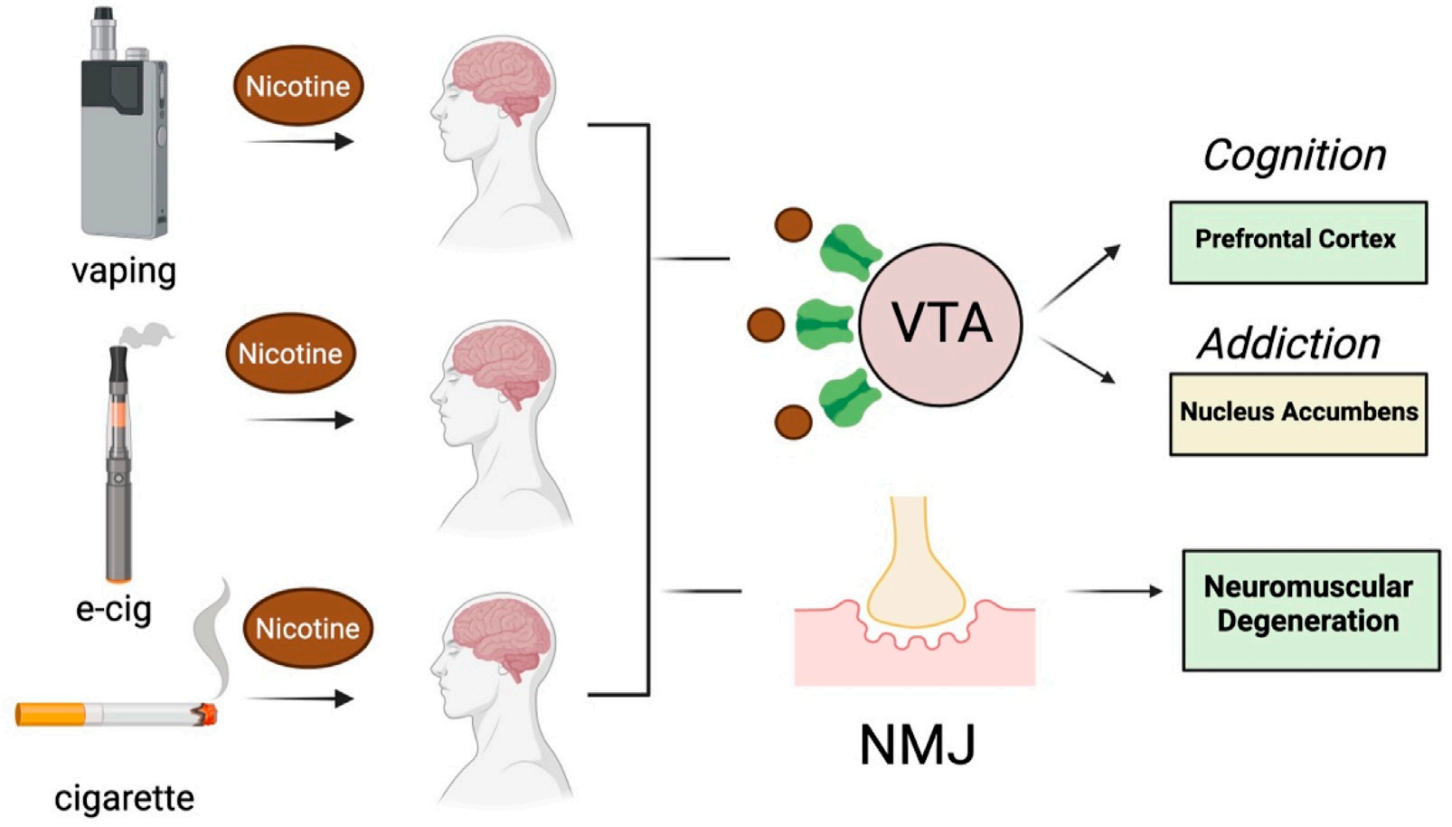
Effect of nicotine on brain and neuromuscular function following exposure to vapors or SHS generated by e-cigarettes and conventional cigarettes (respectively). VTA, ventral tegmentum area, NMJ, neuromuscular junction. Images were created with BioRender.

Nicotine is one of the most abundant toxins in SHS (mg to µg quantities), next to aldehydes and polycyclic aromatic hydrocarbons (Arfaeinia et al., 2023), and is also produced after the heating of tobacco products (*e.g.*, e-cigarettes) (Upadhyay et al., 2023). EC cigarette pods contain approximately 59.2–66.7 mg/mL of nicotine, which is comparable to one pack of 20 conventional cigarettes (Lopez-Ojeda and Hurley, 2024). Nicotine levels in e-cigarette aerosols can range anywhere from 0–50 mg/mL of liquid (Yan et al., 2021; Goniewicz et al., 2018; Prochaska et al., 2022) and are reportedly lower following exposure of individuals to vapors vs SHS (Tattan-Birch et al., 2024). This difference may be explained by the 99% retention of nicotine by vapors following inhalation (Czogala et al., 2014; St Helen et al., 2016). SHS inhaled from conventional cigarettes or e-cigarette vapors is absorbed into the pulmonary circulation where it binds to neuronal nicotinic acetylcholine receptors (nAChRs) that mediate fast neurotransmission in both the central and peripheral nervous system (Wells and Lotfipour, 2023). The inhaled nicotine causes the release of multiple neurotransmitters (*e.g.*, dopamine, norepinepinephrine, acetylcholine, GABA and glutamate) in the reward/addiction pathways and involved in cognition, as well as activation of nicotinic receptors at the neuromuscular junction (Figure 2). Cholinergic receptors are located in several brain regions notably the midbrain tegmentum, the striatum, nucleus accumbens and the ventral tegmentum (VTA). The addictive properties of nicotine are reportedly due to activation of nACHRs in the brain to cause the release acetylcholine and dopamine in the nucleus acccumbens (Tiwari et al., 2020). GABAergic, serotonergic, noradrenergic, and brain stem cholinergic may also mediate the actions of nicotine on the brain. The addictive properties of nicotine may also be related to its activation of both dopaminergic neurons of VTA as well the GABA-ergic neurons (Varani et al., 2018). The activation of nicotinic receptors at the neuromuscular junction by SHS can also cause degeneration, consistent with a key role for smoke exposure causing denervation in patients with chronic pulmonary disease (Kapchinsky et al., 2018). In the CNS, nicotine modulates the reward/addiction pathways and cognition through activation of nAChRs in the mesocortical and mesolimbic dopaminergic (DA) pathways (Ikemoto and Bonci, 2014). The rewarding and cognitive effects of nicotine are mediated through the activation of mesocortical DA receptors in the prefrontal cortex and anterior cingulate cortex, while the activation of DA receptors by nicotine in the nucleus accumbens and amygdala modulate synaptic plasticity and long-term potentiation that are more important in addiction.

Inhaled nicotine can also have long-term effects on brain development, because nicotinic acetylcholine receptors (nAChRs) play a very important role in modulating the release of neurotransmitters during key stages of neurodevelopment (England et al., 2017). Nicotinic receptors regulate critical aspects of brain maturation during the prenatal, early postnatal, and adolescent periods (Dwyer et al., 2009). Nicotine interferes with catecholamine and brainstem autonomic nuclei development during the rodent prenatal period (first and second trimester in humans), alters the neocortex, hippocampus, and cerebellum during the early rodent postnatal period (third trimester in humans) and influences the limbic system and later monoamine-containing neuron maturation during adolescence (Dwyer et al., 2009). SHS exposure during fetal (prenatal) or neonatal (postnatal) brain development can also produce long-term effects on the developing brain to disrupt brain plasticity and overall brain structure (*e.g.*, volume, thinning) to lead to neurodevelopmental disorders (*e.g.*, ADHD, schizophrenia, ASD, and anxiety) (Ou et al., 2024; Herrmann et al., 2008; Colyer-Patel et al., 2023; Greenwood et al., 2024).

Prenatal exposure to nicotine from tobacco products might also produce neurodevelopmental delay through epigenetic changes (Buck et al., 2020; Gould, 2023; Hoang et al., 2024). DNA methylation changes are observed in mothers who are exposed to cigarette smoke during pregnancy (Markunas et al., 2014). Markunas and colleagues (Markunas et al., 2014) showed that DNA methylation is changed in 110 gene regions and notably *FRMD4A*, a gene associated with Alzheimer disease (AD) and nicotine dependence and *CNTNAP2*, a gene associates with neural development, autism spectrum disorder, schizophrenia, and language impairment. Another study (Rauschert et al., 2019) also revealed that tobacco use is associated with differential DNA methylation of both *FRMD4A* and *CNTNAP2*. More recently, Hoang and colleagues (Hoang et al., 2024) reported that *in utero* exposure to environmental tobacco smoke (ETS) alters DNA methylation of both *CNTNAP2* and *FRMD4a* that might persist into adulthood. Collectively, these studies provide evidence that prenatal exposure to nicotine in tobacco smoke can alter brain processes involved in neural development, age-related neurodegenerative conditions like Alzheimer’s disease, and addiction.

Neurotrophins, like nerve growth factor (NGF) and brain-derived neurotrophic factor (BDNF), play important roles in neuronal development, function, and survival during early stages of both the central and peripheral nervous system (Ferraguti et al., 2023). In general, the brain peptide system plays an important role in nicotine addiction and drugs that target this system prevent the activation of reward systems (Bruijnzeel, 2017; Boiangiu et al., 2023). Exposure of the fetus to SHS during pregnancy appears to alter the expression of neuropeptides (BDNF, PCAP) by the region-specific activation of nicotinic receptors (Machaalani et al., 2019). Nicotine also increases brain-derived neurotrophic factor (BDNF) levels in the hippocampus and neocortex. Mice exposed to environmental tobacco smoke (ETS) during the first two postnatal weeks show lower locomotor activity, anxiety-like behavior and corresponding reduced levels of synaptic proteins and BDNF in the cerebellum, striatum and prefrontal cortex (Torres et al., 2018). Exposure to tobacco smoke during various periods of brain development also alters synaptic and neurotrophin levels in different brain regions that appear to last long after early life exposure to SHS.

## 3 Pharmacology and pathology of other SHS chemicals on the brain

As discussed above, smoke from cigarettes or e-cigarettes contains many chemicals at levels comparable to nicotine (*see* Table 1). SHS also contains aldehydes and polycyclic aromatic hydrocarbons (PAHs), as well as lower concentrations of other constituents (Arfaeinia et al., 2023), which have also been detected in e-cigarette vapors or even after the heating (*versus* combustion) of tobacco products (HTP) (Upadhyay et al., 2023; Vivarelli et al., 2022). HTPs are a newer category of tobacco products that generate nicotine and other chemicals, but at lower amounts than conventional cigarettes. Sufficient evidence supports that pre-natal PAH exposure negatively impacts cognitive development with specific regard to child intelligence (Humphreys and Valdes Hernandez, 2022). PAH during childhood and as an adult also was associated with an increase in biomarkers of neuroinflammation found in neurodegenerative diseases like AD and PD (Humphreys and Valdes Hernandez, 2022). There are ethnicity differences in SHS exposure that need to be considered as well. Smoking and SHS exposure accounts for the largest exposure to PAHs of non-Hispanic Whites vs other age-matched ethnic groups as well as older age groups (Gearhart-Serna et al., 2021). This large study indicates that there are vulnerable subpopulations with high PAH intake as a result of different smoking behaviors and potentially other exposures. Prenatal exposure to PAHs might alter mitochondrial copy number as a mechanism to explain its ability to impair neurodevelopment in children (Cao et al., 2020). Cord blood levels of benzo [a]pyrene DNA adducts (marker of PAH exposure) are inversely associated with the development of infants born to pregnant mothers who are exposed to PAHs from a Chinese coal burning power plant (Kalia et al., 2017). Benzo [a]pyrene DNA adducts levels are also negatively associated with BDNF levels in cord blood, suggesting that PAHs might cause neurodevelopmental delay through a DNA damage-mediated mechanism. The PAH concentration in cord blood after exposure to SHS would be expected to be much lower than after exposure of mothers to PAHs from a coal-burning plant.

**FIGURE 3:**
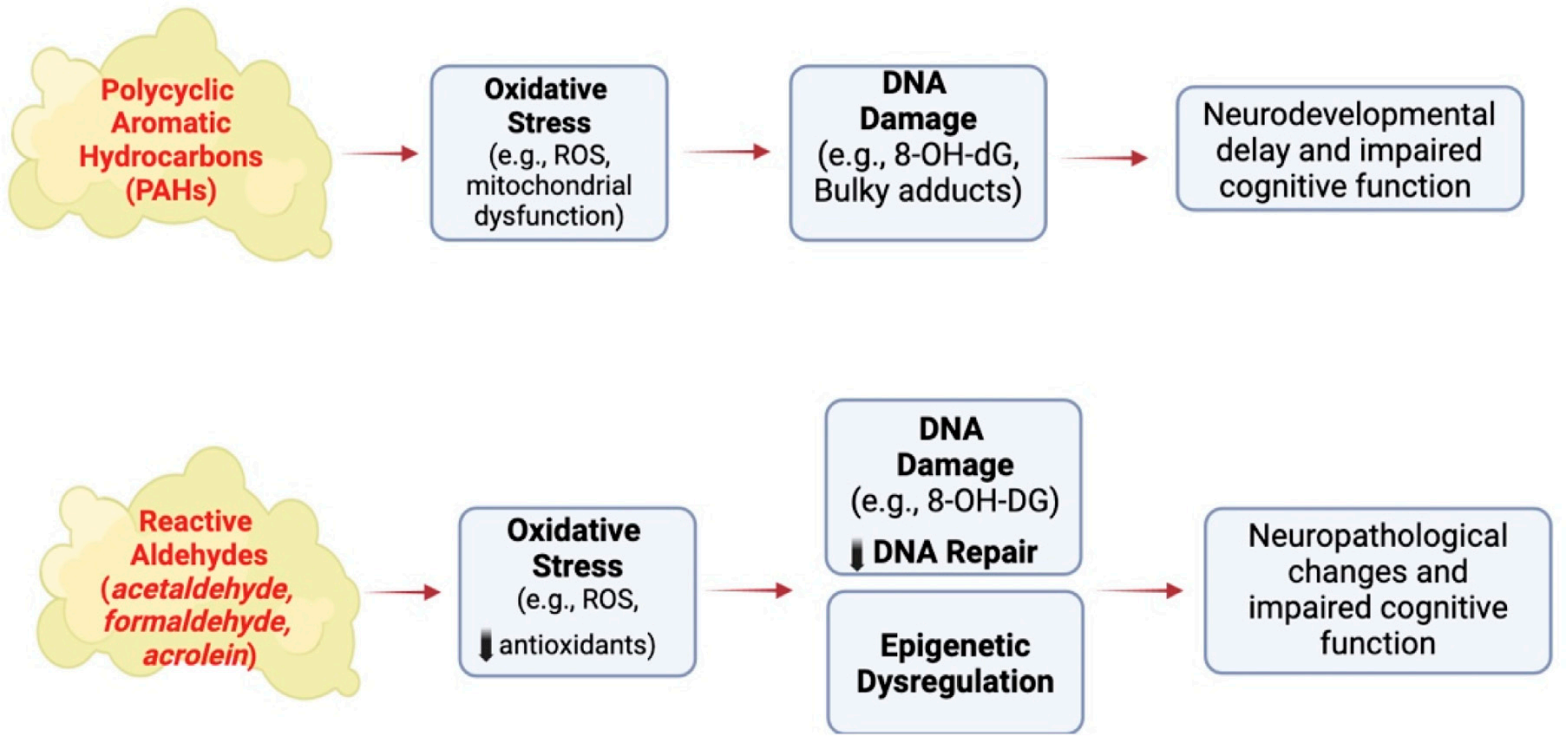
Effect of other SHS chemicals on brain structure and function following exposure to conventional cigarettes. ROS, reactive oxygen species, 8-OH-dG, 8-hydroxy-2′-deoxyguaonsine,. Images were created using BioRender software.

Reactive aldehydes like acetaldehyde, acrolein and formaldehyde are formed during the combustion of tobacco products (Tulen et al., 2022). Acetaldehyde induces cytotoxicity by disrupting mitochondrial function to cause oxidative stress in neural cells (Yan et al., 2022), while acrolein causes DNA damage and oxidative stress in non-neural cells (Bellamri et al., 2022; Hikisz and Jacenik, 2023) and is elevated in the brain of patients with neurodegenerative diseases (Chang et al., 2022). Acrolein and formaldehyde induce Alzheimer-like disease pathology when administered to rodents (Liu et al., 2018; Chen et al., 2022) or primates (Zhai et al., 2018). Adult male rats treated for 3 months with acrolein show neurobehavioral alterations and cognitive impairments that are associated with electrophysiological disturbances (Khoramjouy et al., 2021). Endogenous acrolein plays a significant role in the pathogenesis of various neurodegenerative diseases, including Alzheimer’s disease (Chang et al., 2022), possibly through inducing oxidative stress reducing brain antioxidant levels and activating the MAPK pathway resulting in the hyperphosphorylation of tau and increasing amyloid-β levels, both biomarkers of neuropathology (Dhapola et al., 2023; Jallow et al., 2024). Thus, environmental exposure of humans to tobacco smoke and endogenous antioxidant levels could be important risk factors for the developing as well as adult brain (Chang et al., 2022) (Figure 3).

Formaldehyde (FA) is as environmental contaminant with toxic potential that also serves as an indispensable and thus normal physiological metabolite in the healthy brain, where it is hypothesized to regulate learning and memory via the *N*-methyl-D-aspartate receptor (Ai et al., 2019). FA is also a product of various metabolic pathways that participate in the one-carbon cycle, which provides carbon for the synthesis and modification of bio-compounds, such as DNA, RNA, and amino acids (Li et al., 2021). Endogenous FA plays a role in epigenetic regulation by regulating the methylation and demethylation of DNA, histones, and RNA (Li et al., 2021). At high levels, FA can pose a significant threat to genomic stability (Reingruber and Pontel, 2018), DNA repair (Nadalutti et al., 2021; Weng et al., 2018; Tang et al., 2022) and impede transcription, with negative physiological consequences (Mulderrig et al., 2021), through epigenetic alteration (Li et al., 2021), including neuronal and endothelial damage, (Chen et al., 2024). Notably, impaired memory is observed in mice with elevated endogenous FA, induced by knock-out of the gene coding for aldehyde dehydrogenase-2, a key mitochondrial enzyme for the effective metabolism of alcohol and acetaldehyde (Ai et al., 2019). Exposure to high levels of FA by inhalation (3.0 mg/m^3^) impairs cognitive function, including memory, in humans, causing neuronal damage and oxidative stress in the cerebellum of experimental animals, and inducing the misfolding of neuronal tau and related proteins *in vitro* (Rana et al., 2021). The balance between genotoxins and benign metabolites is presumed to depend on concentration, localization, pH and redox state, features that are or potentially altered during disease progression (Hopkinson and Schofield, 2018).

## 4 Pathway changes associated with cellular damage and inflammation in brain

A systematic review of the effects of active and passive smoking of conventional cigarettes, electronic cigarettes and tobacco heating products indicated that active and passive smoking induce oxidative stress and inflammatory responses in peripheral tissues (Kopa-Stojak and Pawliczak, 2024; Zieba et al., 2024), but the nervous system was not examined (Kopa-Stojak and Pawliczak, 2024). Oxidative stress-induced DNA damage and inflammation are also emerging as key triggers of dementia and related neurological disorders (Houldsworth, 2024; Giri et al., 2024; Neven et al., 2024; Firdous et al., 2024), as well as neurodevelopmental disorders (Qing et al., 2023; Lubrano et al., 2024; Xu et al., 2024). Since 90% of neurodegenerative diseases (*e.g.*, MCI, dementia) are sporadic, this suggests that environmental factors like tobacco smoke might play an important, but undefined, role in their etiology (Ourry et al., 2024; Pandics et al., 2023). Early life exposure to SHS may also be an important risk factor for dementia (Chen, 2012; Zhou and Wang, 2021; Wan et al., 2024), as well as neurodevelopmental disorders (Hall et al., 2016; Julvez et al., 2021; Mukhopadhyay et al., 2010; Wade et al., 2023). Studies with animal models suggest that the effect of SHS on the brain may be due to increased oxidative stress and inflammation during brain development, leading to increased brain cell apoptosis in adulthood (Vivarelli et al., 2024; Raber et al., 2021; Raber et al., 2023; Lopes et al., 2023). Short-term exposure of 2-month-old mice (6h/day x 5 days/wk x four or 8 weeks) to a mixture of sidestream/mainstream cigarette smoke impairs brain insulin signaling and induces the accumulation of neuropathological proteins (Deochand et al., 2015; Deochand et al., 2016). Shorter durations of mainstream/sidestream smoke (1h/day x 1 month) induces lipid peroxides, DNA damage, and tau dysregulation (tau isomers, phosphotau) in the brain of neonatal mice (La Maestra et al., 2011), markers of neuropathology frequently observed in MCI and patients with dementia (Lovell and Markesbery, 2007; Simpson et al., 2016; Wirz et al., 2014). Longer exposures of 2-month-old rats or 3-month-old APP/PS1 transgenic mice to sidestream cigarette smoke (1h/day x 5 days/wk x two or 4 months) induces tau and amyloid pathology like that reported in MCI and patients with dementia (Ho et al., 2012; Moreno-Gonzalez et al., 2013). These studies strongly suggest that SHS increases the risk of developing MCI and dementia by perturbing brain metabolism (*i.e*., insulin signaling, oxidative stress) and the accumulation of neuropathological proteins (*i.e*., tau, amyloid). SHS induces a distinct brain metabolic profile characterized by oxidative stress (Raber et al., 2021; Raber et al., 2023; Neal et al., 2016) and inflammation (Lopes et al., 2023; D et al., 2019; Chan et al., 2020). The cortex and hippocampus in the brains from the offspring of female C57BL/6 mice exposed to air or SHS (50 μg/m^3^; 5h/day, 5 days/week for 5 weeks and 2 days) were examined by untargeted metabolomics. Insulin signaling, which regulates an abundance of metabolic proteins, is altered in the hippocampus of the offspring exposed throughout development to SHS. An increase in glutathione-S-transferase is also detected, and a trend towards increased glutathione reductase activity, increases GSSG, and a decreased GSH/GSSG ratio is observed. In a systematic review of the literature, it was also reported that cigarette smoking (active and passive) induces oxidative stress and an inflammatory response in peripheral (*i.e.*, non-neurological) tissues (Kopa-Stojak and Pawliczak, 2024; Kanithi et al., 2022; Prasad and Bondy, 2022; Mukharjee et al., 2020). Thus, exposure to SHS (passive smoking) on the brain and non-neurological tissues induces both oxidative stress and inflammation.

**FIGURE 4:**
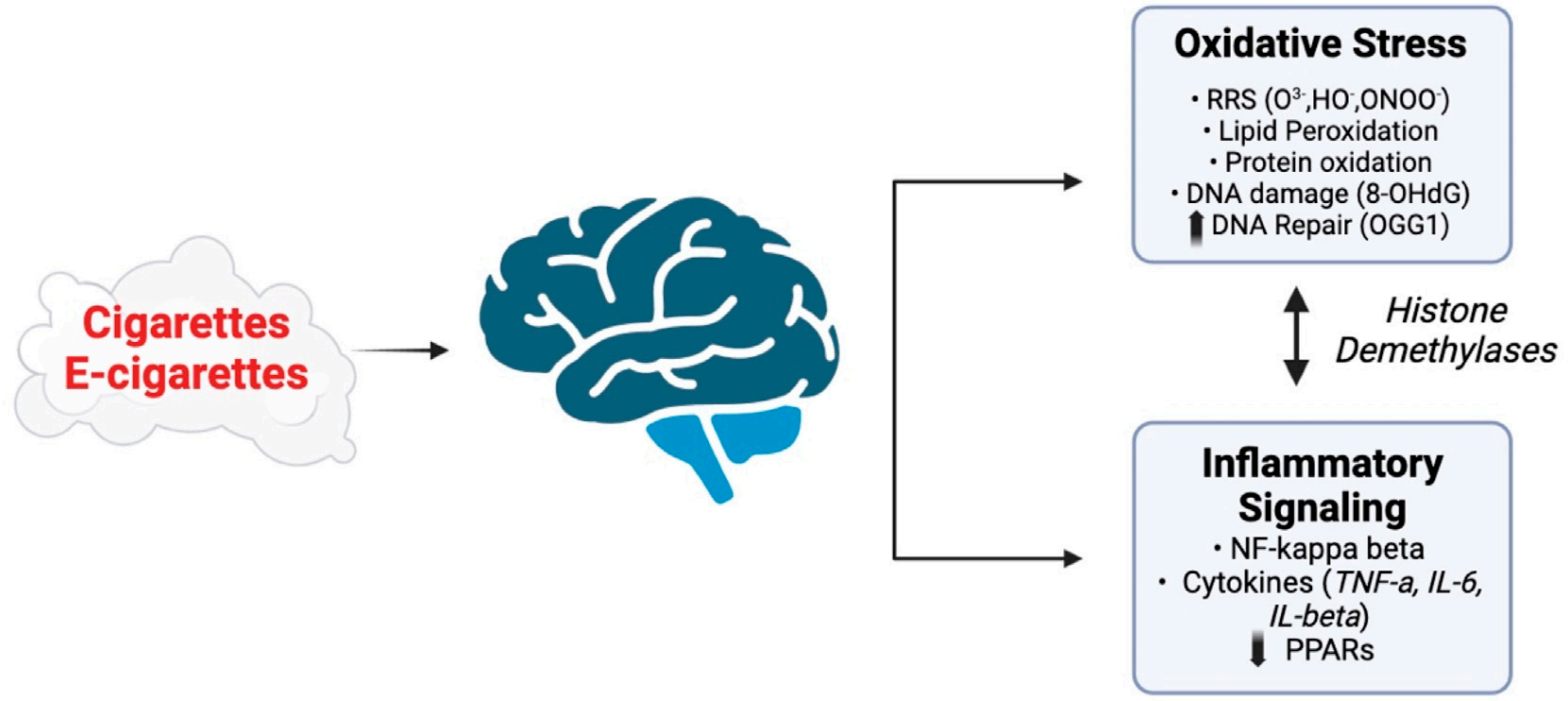
Potential pathways affected by exposure of the CNS to vapors or SHS generated by e-cigarettes and conventional cigarettes. Adapted from Vivarelli et al. (2024), Arfaeinia et al. (2023), Addissouky et al. (2024), and Auschwitz et al. (2023). Images were created using BioRender software.

A multi-center study of six European countries that examined the relationship between early life environmental exposures and child cognitive function found that ETS exposures adversely and cross sectionally associate with cognitive function (Julvez et al., 2021). In human studies, maternal exposure to SHS is also closely linked to small brain size and changes in brain structure that associated with a higher risk of cognitive impairments (Colyer-Patel et al., 2023; Greenwood et al., 2024; Chan et al., 2020) and psychotic experiences (Wang D. et al., 2024). SHS also induces oxidative stress and inflammation in the developing brain (D et al., 2019; Chan et al., 2020; Lobo Torres et al., 2012; Mohamed et al., 2022; Church et al., 2020). Acute exposure to SHS on postnatal day 18 increased GST activity and malondialdehyde (MDA levels in the hippocampus, GPx and SOD activity in the prefrontal cortex and GST activity and MDA levels in the striatum and cerebellum of postnatal mice (Lobo Torres et al., 2012). Three hours later, SOD activity and MDA levels increased in the hippocampus and the activity of all enzymes decreased in the prefrontal cortex. This study shows that SHS induces oxidative stress by perturbing antioxidant enzymes in distinct brain regions during early brain development like that reported in older animals. Thus, oxidative stress appears to be an early event following exposure to ETS or SHS from tobacco products. Pregnant mice were exposed to e-cig vapor (2.4% nicotine) from GD five until postnatal day 7 (PD7) and the brain of mice at PD7 and PD 90 examined for reactive oxygen species (ROS) and pro-inflammatory cytokines (Archie et al., 2023). E-cig vapor reduced antioxidant marker expression and increased the expression of pro-inflammatory and cytokine markers in the PD7 brain, but not the PD 90 brain. Pregnant mice were also exposed daily to e-cigarette chemicals (propylene glycol, vegetable glycol) and 16 mg/mL of nicotine for 3 h/d, 7 days a week from gestational day (GD) 0.5 until GD 17.5 (Church et al., 2020). Male and female offspring of e-cigarette exposed mice had lower scores on the novel object recognition task and reduced inflammatory markers in the diencephalon (IL-4, IFNγ) and hippocampus (IFNγ; females only). This experimental study demonstrates that e-cigarette vapors can also persistently alter the neuroimmunology and behavior following maternal exposure. Thus, oxidative stress and inflammatory markers are also increased in the brain of mice after *in utero* exposure to SHS from both conventional cigarettes and e-cigarettes (Figure 4).

## 5 MCI, neurodegeneration, and other neurological disorders

As described earlier, there is growing evidence that non-smokers exposed to SHS are at an increased risk of developing MCI (Chen, 2012; Llewellyn et al., 2009; Yolton et al., 2005; Murphy et al., 2020; Akhtar et al., 2013; Cataldo et al., 2010; Chang et al., 2012) and dementia (Chen, 2012; Cataldo et al., 2010; Chang et al., 2012), as well as neurodevelopmental disorders (Ou et al., 2024; Chan et al., 2020; Pagani, 2014). Exposure to SHS increases the risk for dementia among individuals who never smoked (Zhou and Wang, 2021; Wan et al., 2024; He et al., 2020) and it is 2–6 times more toxic and tumorigenic to humans than mainstream smoke (Schick and Glantz, 2005; Akhtar et al., 2013). Cholinergic dysfunction of the nucleus basalis of Meynert (NBM) is hypothesized to be an important factor for the increased risk of AD (Slotkin et al., 2015; Slotkin et al., 2019; Toro et al., 2008). Chronic exposure to nicotine through smoking may lead to atrophy of cholinergic input areas of the basal forebrain. Chronic exposure to nicotine through smoking disrupts the functional connectivity between the NBM and precuneus in MCI patients (Qiu et al., 2022). The ability of cigarette smoke to disrupt the connectivity in both non-smokers and those with MCI suggests that exposure to cigarette smoke disrupts cognition. Yet, 6 months of transdermal nicotine administration (16 mg/day) to MCI patients improves primary and secondary cognitive measures of attention, memory, and mental processing (Heffernan and O'Neill, 2013). These studies demonstrate that exposure to nicotine through cigarette smoke can either disrupt cholinergic function or be protective. Early life exposure to nicotine and other SHS constituents might be the key to understanding how the toxins in cigarette smoke induce their long-term effects on learning and memory, executive function, and the reward circuitry (Hall et al., 2016; Cauley et al., 2018; Ponzoni et al., 2020).

**FIGURE 5:**
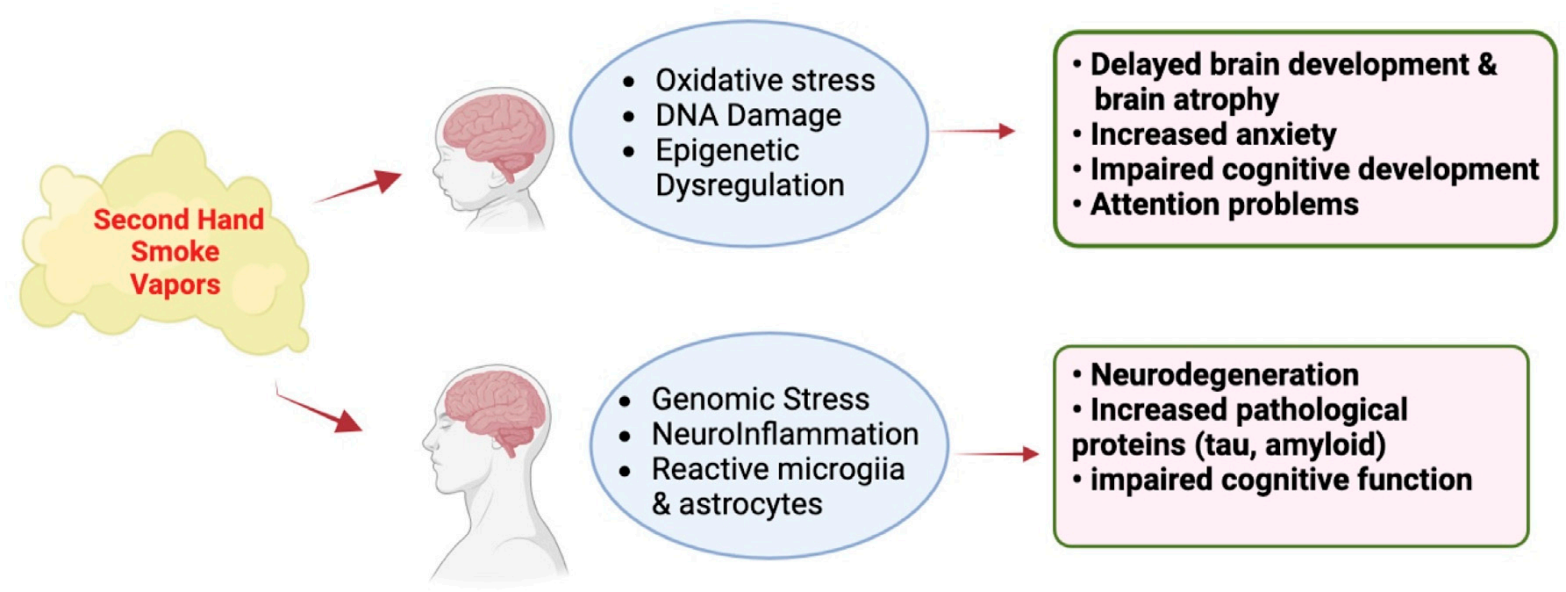
Effect of vapors or SHS generated by e-cigarettes and conventional cigarettes (respectively) on the developing and mature CNS. Adapted from Merecz-Sadowska et al. (2020), Prasedya et al. (2020), Chan et al. (2020), and Garza et al. (2023). Images were generated using BioRender software.

Base excision repair (BER) is the primary cellular pathway for repairing oxidative DNA damage *(e.g.*, 8-oxo-deoxyguanosine, 8-oxodG) (Oka et al., 2021) that is reportedly impaired in both MCI individuals and those with AD (Chang et al., 2022; Cherbuin et al., 2024). Furthermore, the brain of dementia subjects also exhibits activation of the DNA damage response (DDR) pathway, inflammatory changes and cellular senescence (Schwab et al., 2021). Exposing mice for 4 months to active (La Maestra et al., 2011) or passive (Moreno-Gonzalez et al., 2013) cigarette smoke induces oxidative stress, DNA damage and neuropathology in mice. Chronic exposure of mice to SHS (90% side stream, 10% mainstream smoke x 2.8 h/day x 7 days/wk x 10.4 mos) induces dark, shrunken cells, hippocampal thinning, and the presence of activated astrocytes and prominent 8-oxoG staining in the prefrontal cortex (PFC) and hippocampus (HIPP) (Raber et al., 2021; Lopes et al., 2023). 8-oxoguanine DNA glycosylase (Ogg1) staining is also reduced in the PFC and CA3 hippocampal neurons of SHS chronically exposed mice. Apurinic/apyrimdinic endonuclease (Ape1) staining is more prominent in the PFC and the HIPP in SHS chronically exposed mice. These studies demonstrate that oxidative DNA damage (8-oxoG) is elevated and oxidative DNA repair (Ogg1 and Ape1) is altered in the brain of SHS exposed mice, as well as activation of reactive astrocytes. The percentage of 8-OHdG-labeled cells in the CA1 region of the hippocampus is associated with performance in the novel object recognition test, consistent with urine and serum levels of 8-OHdG serving as a biomarker of cognitive performance in humans. Therefore, SHS induces both oxidative DNA damage and repair, as well as inflammation as possible underlying mechanism(s) of the behavioral and cognitive function and metabolic changes that were observed in chronically exposed mice (Raber et al., 2021). These findings suggest that human exposure to cigarette smoke induces oxidative stress, genomic stress (i.e., DNA damage and repair) and enhances neuroinflammation like that in age-related neurodegenerative diseases (Simpson et al., 2016; Shadfar et al., 2023) (Figure 5).

With regard to neurodevelopmental disorders, a recent META analysis involving 54 studies revealed associations between SHS exposure and the risks of developing attention deficit hyperactivity disorder (ADHD) and learning disabilities (LD) (Ou et al., 2024). There is also an association between cotinine exposure and ADHD. Consistent with the increased ADHD risk, prenatal SHS and postnatal maternal distress alter the efficiency of the cingulo-opercular (CO) network, which is involved in task control and executive function (Freedman et al., 2020). In addition to SHS, third hand smoke, consisting of residual tobacco smoke pollutants that remain on surfaces and in dust after tobacco has been smoked, which are re-emitted into the gas phase or react with oxidants and other compounds in the environment to yield secondary pollutants and include nicotine, three-ethenylpyridine (3-EP), phenol, cresols, naphthalene, formaldehyde, and tobacco- specific nitrosamines that have detrimental effects on the developing brain (Matt et al., 2011). Third hand smoke can be inhaled through inhalation, ingestion, or dermal uptake from the air, dust, and from surfaces. Consistent with the human data, third hand smoke for 4 weeks in mice increased inflammatory cytokines in plasma and increased epinephrine and aspartate aminotransferase, a biomarker of liver damage (Adhami et al., 2017). These detrimental effects are more pronounced when the mice were chronically exposed to third hand smoke for 8, 16, and 24 weeks. With longer third hand smoke exposure, mice become hyperglycemic and hyperinsulinimic, indicating an important role for impaired insulin sensitivity after third hand smoke exposure. Pre- and post-natally, there can also be a combination of SHS and third hand smoke.

## 6 Conclusion

**FIGURE 6:**
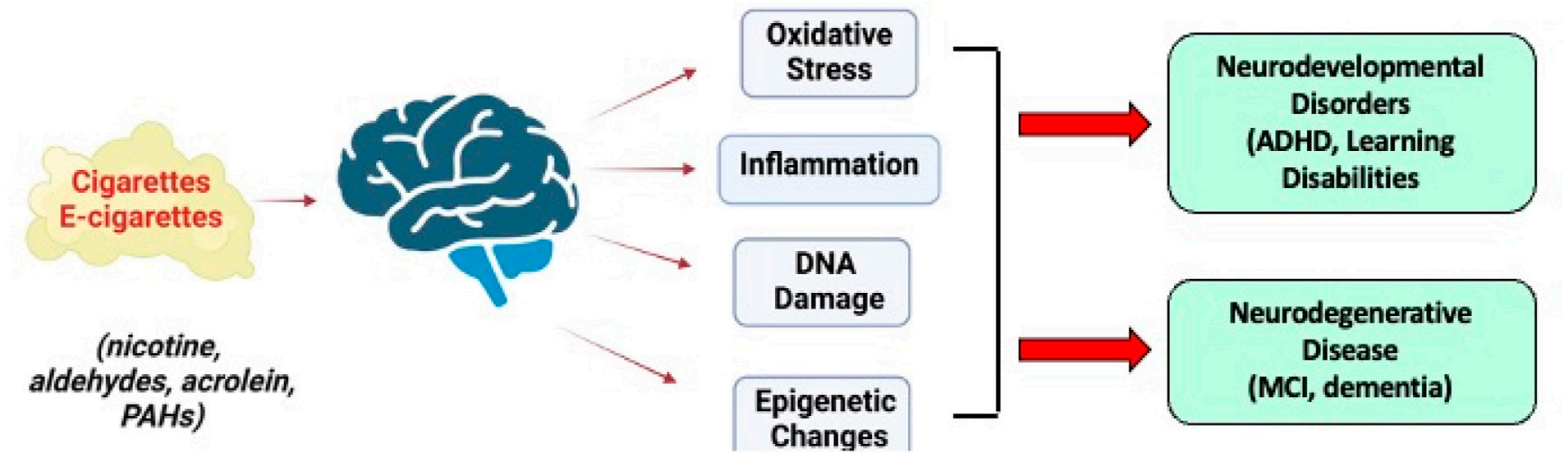
Long-term effects of SHS constituents from conventional and e-cigarettes on the brain and their role in neurodevelopmental disorders and neurodegenerative disease. Images were created with BioRender.

The above studies of SHS exposure in animals and humans demonstrate that the brain is a key target of nicotine and other constituents following exposure to tobacco products. Early life exposure to SHS disrupts brain development to increase the risk for neurodevelopmental disorders (ADHD, learning disabilities). On the other hand, exposure of the mature brain to SHS is considered a risk factor mild cognitive impairment and dementia. A common target for SHS in the developing and adult brain is oxidative stress, inflammation responses and genomic stress that might be responsible for triggering both neurodevelopmental disorders as well as dementia (Figure 6). Given the world-wide exposure of pregnant women, children and adults to SHS, additional research will be required to pinpoint the mechanism by which SHS is a risk factor for both neurodevelopmental and neurodegenerative disorders with the goal of protecting the most vulnerable to these environmental exposures.
